# Dynamic changes in postpartum autonomic nervous system in normal mice: a pilot study

**DOI:** 10.7717/peerj.21142

**Published:** 2026-05-18

**Authors:** Kie Shimizu, Sayaka Kuze-Arata, Takefumi Kikusui, Kazutaka Mogi

**Affiliations:** 1 Department of Animal Science and Biotechnology, Azabu University, Sagamihara, Kanagawa, Japan; 2 Department of Veterinary Nursing and Health Science, Azabu University, Sagamihara, Kanagawa, Japan

**Keywords:** Autonomic nervous activity, Electrocardiogram, Heart rate variability analysis, Longitudinal study, Dynamic changes, Mouse

## Abstract

Female mammalian physiology changes dynamically during parturition. Autonomic nervous activity plays a key role in normal parturition and is influenced by the health and mental state of mothers; however, few longitudinal studies have tracked individual changes in autonomic nervous activity during the 24 h surrounding parturition. We conducted a longitudinal study in awake mice using a telemetry device to record electrocardiograms before, during, and 24 h after parturition. Half of the mice gave birth and reared their offspring normally (normal group), whereas the remaining mice experienced parturition failure, neglect, or cannibalism/infanticide (abnormal group). We compared autonomic nervous activity between normal and abnormal groups using time-domain analysis of heart rate variability. At 24 h after parturition, normal mice showed a significant increase in the following heart rate variability parameters: standard deviation of the intervals between two consecutive R-wave peaks (RR intervals) in electrocardiograms, root-mean-square of successive differences, and coefficient of variation of RR intervals. Abnormal mice did not show these changes, and these parameters remained lower than those of normal mice. These findings suggest that autonomic nervous activity may be associated with adaptation from pregnancy to lactation periods. Detecting dynamic changes in autonomic nervous activity within the 24 h after parturition may be useful for characterizing normal mothers.

## Introduction

In mammalian females, physiological functions undergo dynamic changes throughout pregnancy, parturition, and the postpartum period (Costantine, 2014; Reyes-Lagos et al., 2019). These changes involve autonomic nervous activity, which regulates cardiovascular and metabolic adaptations. Notably, sympathetic and parasympathetic activity decreases during pregnancy (Reyes-Lagos et al., 2019; Brown et al., 2021) and increases postpartum (Nagel et al., 2016; Musa et al., 2017) compared to the non-pregnant state. The autonomic nervous system plays an essential role in normal parturition, with the sympathetic nervous system regulating myometrial contractility and the vagus nerve mitigating inflammation (Brauer & Smith, 2015; Reyes-Lagos et al., 2019). Additionally, hormonal secretions leading up to parturition, such as estrogen and progesterone secretions, likely influence autonomic nervous activity (Reyes-Lagos et al., 2019; Kodogo, Azibani & Sliwa, 2019; Georgescu, 2023). Although previous reports have compared autonomic nervous activity before and after parturition in humans (Papadopoulos et al., 2021; Uçar, Bülbül & Yıldız, 2022) and horses (Nagel et al., 2014; Felici et al., 2023), few longitudinal studies have tracked individual changes in autonomic nervous activity during the 24 h surrounding parturition.

The health and mental state of mothers during pregnancy and parturition can also affect autonomic nervous activity. For example, compared to that in normal pregnancies, human autonomic nervous activity decreases with conditions such as psychiatric disorders (Shea et al., 2008; Mizuno, Tamakoshi & Tanabe, 2017), preterm parturition (Reyes-Lagos et al., 2019), and cesarean section (Papadopoulos et al., 2021; Uçar, Bülbül & Yıldız, 2022). Additionally, stressed mothers exhibit lower autonomic nervous activity during the postnatal period (Parisi et al., 2024), suggesting that maternal autonomic activity fluctuates with internal and external factors. Abusive or neglectful mothers are more likely to experience mental disorders (Ben David, 2021) and high parental stress (Martins, Matos & Sani, 2023), and abnormal maternal behaviors may lead to reduced autonomic activity.

Most studies on autonomic nervous activity in pregnant mice have been conducted under anesthesia (El Khoury et al., 2013; Kasahara et al., 2021; Khandoker et al., 2022). However, our previous studies demonstrated the feasibility of longitudinally measuring autonomic nervous activity in awake C57BL/6J mice from pregnancy to the postnatal period (Shimizu et al., 2024), with no functional abnormalities in electrocardiogram (ECG) readings (Moreth et al., 2014).

In this study, we employed the same method, as well as heart rate variability (HRV) analysis, to measure autonomic nervous activity in normal and abnormal mice (including parturition failure, neglect, and cannibalism/infanticide), which were classified based on observed behaviors from gestation day (GD) 14 to postnatal day (PD) 21 (Table 1). Specifically, we measured autonomic nervous activity in normal and abnormal groups during the 24 h surrounding parturition, enabling us to track individual differences. Our aim was to investigate the dynamic characteristics of autonomic nervous activity by comparing normal and abnormal states, and to examine whether autonomic nervous activity can predict abnormal parturition and maternal behaviors.

**Table 1:** Information on individual mice. This table includes data on individual mice, such as the number of pups raised, start time of parturition, light phase/dark phase, time after lights-on (h) and birth date of litters.

**Number**	**Number of pups raised**	**Parturition failure Cannibalism/infanticide** **Neglect**	**Start time of parturition**	**Light phase/ Dark phase**	**Time after lights-on (h)**	**Birth date**
1	4		22:12	Dark	16.2	GD18
2	4		22:23	Dark	16.4	GD18
3	4		23:37	Dark	17.6	GD18
4	4		2:20	Dark	20.3	GD18
5	0	Parturition failure	14:40	Light	8.7	GD18
6	0	Neglect	21:13	Dark	15.2	GD18
7	3	Cannibalism/infanticide (PD13)	12:31	Dark	6.5	GD19
8	0	Parturition failure	16:32	Light	10.5	GD21
9	3	Cannibalism/infanticide (PD1)	22:14	Dark	16.23	GD18
10	4		19:07	Dark	13.12	GD18

## Materials & Methods

### Animals

Ten pregnant C57BL/6J mice at GD14 were used in this study. Mice were obtained from Japan Clea Co., Ltd. (Yokohama, Japan) and housed individually in a polymethylpentene cage (17.5 × 24.5 × 12.5 cm: Japan Clea Co., Yokohama, Japan) for approximately one month. All animals were obtained from the same supplier within the same period and maintained under identical housing conditions to minimize background variability. For environmental enrichment, corn cob (Shepherd’s Cob, Shepherd Specialty Papers, TN, USA) was used for bedding and Palmas αN (Nabebayashi Fuji Science Co., Nagano, Japan) for nesting. Solid food (Certified Diet CRF-1: Oriental Yeast Co., Japan) in a feeder basket (CL-2802: Japan Clea Co., Yokohama, Japan) and water in a bottle (CK-200: Japan Clea Co., Yokohama, Japan) were provided *ad libitum*. Room conditions were maintained at 22–24 °C with a 12-h light-dark cycle, using red light during the dark phase (18:00–06:00). Mouse checks and data collection were performed daily between 07:30 and 08:30. For each litter, the date of birth was designated as PD0. To minimize ECG noise caused by interactions among multiple pups, litter size was culled to two males and two females at PD0. The pups not required for the experiment were euthanized by hypothermia in a −20 °C freezer on PD0, followed by cervical dislocation. Cages were changed once a week. When the cage was changed, half of the bedding and nesting material was replaced with new material, and the remaining half was retained. At the end of the observation period (PD21), between 10:00 and 16:00, all dams and their litters were euthanized by an overdose of isoflurane anesthesia (5% isoflurane for 10–15 min) followed by cervical dislocation. This study was approved by the Animal Experiment Committee of Azabu University (Approval Number: 210319-30).

### Behavioral grouping

Home-cage behavior was recorded using a drive recorder (TS-DP250A-32G, Transcend) mounted on the side of the cage and recorded at 720-p resolution and 30 frames per second, as previously described (Shimizu et al., 2024). Mice were classified into normal and abnormal groups based on parturition and pup survival status, and assessed by continuous home-cage video monitoring and daily morning checks. The onset of parturition was defined as either the appearance of bedding material adhering around the vaginal opening owing to the presence of vaginal fluid, or the appearance of a pup’s head near the vagina (see Video S1 for typical signs). The normal group comprised mice that delivered without parturition failure and successfully raised four pups from PD0 to PD21. The abnormal group comprised mice exhibiting parturition failure, neglect (failure to care for or raise pups post-birth), or cannibalism/infanticide. Parturition failure included cases in which parturition did not progress after delivery of the first pup or failed to initiate, as well as cases in which pups were delivered but showed no spontaneous limb movements or mouth opening immediately after delivery, based on home-cage video monitoring. Cannibalism/infanticide was assessed by reviewing video recordings when pups were found partially eaten or missing during the daily morning check. Cases in which the dam was observed actively eating pups were classified as cannibalism/infanticide. Because the pups were often covered by nest material, their viability at the onset of this behavior could not be confirmed, and the possibility of infanticide could not be excluded. These conditions were analyzed collectively as a single abnormal group because of the small sample sizes for each condition.

### Surgery

Telemetry implantation was performed as described previously (Shimizu et al., 2024). Briefly, pregnant mice (24.5–32.6 g) at GD14 were anesthetized using isoflurane, and telemetry devices (3 g, MT10B, ADInstruments) were implanted on the ventral side (Fig. S1). The negative lead was connected near the sternocleidomastoid muscle, and the positive lead was connected near the xiphoid process. After surgery, recovery was assessed based on home-cage video monitoring. As the negative lead was implanted in the sternocleidomastoid muscle, the ability to lift the head to drink from the water bottle was used as an indicator of recovery. In this study, ECG data were recorded only after the animals could lift their heads to drink, which typically occurred approximately two days after surgery. In this protocol, no analgesics were administered because of their potential effects on fetuses and behavior (Thiele et al., 2015; Dean et al., 2016; Bauer et al., 2021; Elam et al., 2022). Therefore, animals were carefully monitored during the postoperative period. Respiration, spontaneous movement, food intake, and general activity were confirmed by direct observation and by reviewing the home-cage video recordings. All mice showed normal escape responses to handling and maintained normal feeding and drinking behavior. No animals showed signs that required humane endpoint intervention. The sampling rate was set to 1 kHz from GD17 to PD21 to prevent excessive data accumulation.

### Data analysis

All ECG data were reviewed to remove noise segments, as per previous reports (Shimizu et al., 2024). Data were analyzed using LabChart software (Version 8.0, ADInstruments). Heart rate, RR interval (distance between each heartbeat), and HRV were evaluated using the time-domain parameters in the HRV analysis module (ADInstruments). To compare autonomic nervous activity during pregnancy, parturition, and postpartum periods, 1-h ECG data were extracted from each animal (Fig. S2A and Video S2). Owing to noise caused by muscle contractions during parturition (Fig. S2B), 1-h ECG data measured from 1 h before delivery of the first pup were used to represent the parturition period (Fig. S2C). Similarly, data from the same time point 24 h before and 24 h after parturition represented the pregnancy and postpartum periods, respectively (Figs. S2D and S2E). The start time of parturition, the light or dark phase during the recording period, and the time after lights-on were summarized for each animal in Table 1. The time-domain parameters, considered more stable than frequency-domain parameters (Kuss et al., 2008), included the standard deviation of RR intervals (SDRR, reflecting overall autonomic activity), root-mean-square of successive differences (RMSSD, reflecting parasympathetic activity), and coefficient of variation of RR intervals (CVRR, indicating relative parasympathetic to sympathetic activity). The RR interval range was set to 50–250 ms, and complexity, which indicates how closely detected beats conform to the QRS complex, was set between 0 and 9.8.

### Quantification and statistical analysis

Statistical analyses were performed using JMP (Ver. 13, SAS Institute Inc.). The Student’s *t*-test was used to compare the parturition duration and the number of pups born between groups, with results expressed as the mean ± standard error of the mean. All HRV data were analyzed using a two-way repeated measures analysis of variance (ANOVA). The two ANOVA factors of period (24 h before parturition, parturition, and 24 h after parturition) and group (normal and abnormal), were based on individual hourly averages. *Post hoc* tests were conducted using the Bonferroni method. A Bayesian logistic regression model with a compound confluent hypergeometric prior distribution (α = 0.5, β = 2, and s = 0) was constructed using JASP (Ver. 0.19.1, JASP Team).

## Results

### Grouping of dams

Half (five) of the mice underwent normal parturition and raised offspring from PD0 to PD21. However, five mice exhibited abnormal parturition and maternal behaviors, including parturition failure (*n* = 2), neglect (*n* = 1), and cannibalism/infanticide (*n* = 2). Thus, the mice were categorized into two groups: “normal” and “abnormal”. Most parturition events in both groups occurred during the dark phase, except in three mice from the abnormal group, where parturition began during the light phase (Table 1).

### Comparison of parturition characteristics between groups

**Figure 1:**
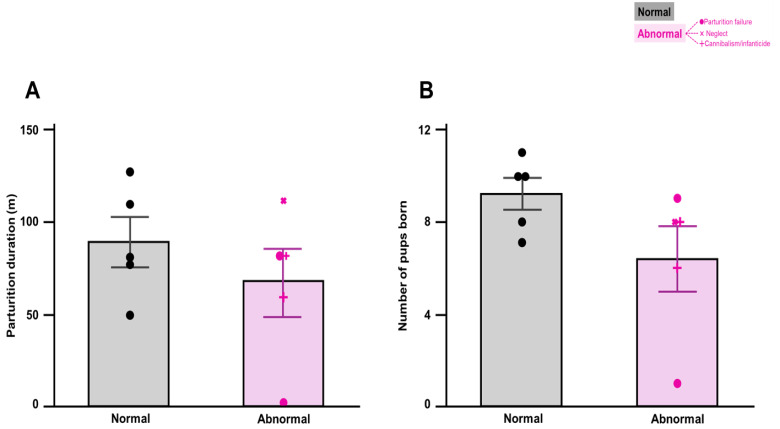
Duration of parturition and number of pups born. (A) Duration of parturition from the first to the last pup. No significant difference was found between normal (*n* = 5) and abnormal (*n* = 5) groups (Student’s *t*-test) The columns represent the mean (±standard error of the mean). (B) Number of pups born. No significant difference was found between normal (*n* = 5) and abnormal (*n* = 5) groups. Student’s *t*-test, Vetrival bars represent the mean (±standard error of the mean).

No significant difference was observed between normal and abnormal groups in the duration of parturition, defined as the time required to deliver all offspring (Student’s t-test: t(8) = 1.0, *p* = 0.36; Fig. 1A). Additionally, no differences were observed in the total number of pups born, including live and stillborn pups (Student’s t-test: t(8) = 1.0, *p* = 0.36; Fig. 1B).

### Comparison of ECG data between groups

The average heart rate (BPM: beats per minute) showed no significant effects of period or group, and no significant interaction between period and group (Period: F[2, 16] = 2.8, *p* = 0.09, Group: F[1, 8] = 3.3, *p* = 0.11, Period × Group: F[2, 16] = 2.8, *p* = 0.14, repeated measures two-way ANOVA; Fig. S3A). The time between two R-waves in the ECG (RR interval) showed no significant main effects of period or group, and no significant interaction between period and group (Period: F[2, 16] = 3.8, *p* = 0.07, Group: F[1, 8] = 2.0, *p* = 0.09, Period × Group: F[2, 16] = 3.1, *p* = 0.17, repeated measures two-way ANOVA; Fig. S3B).

### Comparison of HRV parameters between groups

**Figure 2:**
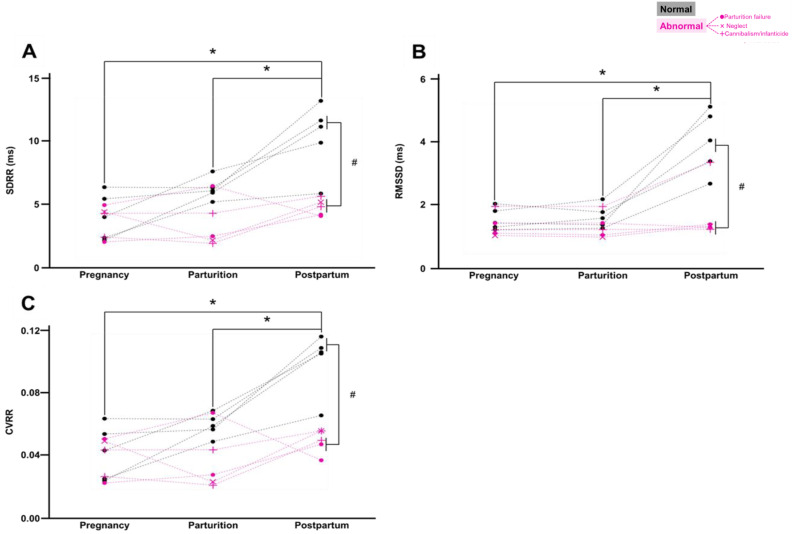
HRV analyzed using time-domain parameters. (A) SDRR (ms) across pregnancy, parturition, and postpartum periods. The normal group (*n* = 5) is shown in black, and the abnormal group (*n* = 5) is shown in red (□: parturition failure , ×: neglect, +: cannibalism/infanticide). Two-way repeated measures ANOVA with Bonferroni test. *p < 0.05 indicates differences between periods in the normal group. #*p* < 0.05 indicates differences between normal and abnormal groups. (B) RMSSD (ms) across pregnancy, parturition, and postpartum periods. Normal group (*n* = 5) is shown in black, and abnormal group (*n* = 5) in red( □: parturition failure, ×: neglect, +: cannibalism/infanticide). Two-way repeated measures ANOVA with Bonferroni test. *p < 0.05 indicates differences between periods in the normal group. #*p* < 0.05 indicates differences between normal and abnormal groups. (C) CVRR across pregnancy, parturition, and postpartum periods. Normal group (*n* = 5) is shown in black, and abnormal group (*n* = 5) in red (□ : parturition failure, ×: neglect, +: cannibalism/infanticide). Two-way repeated measures ANOVA with Bonferroni test. *p < 0.05 indicates differences between periods in the normal group. #*p* < 0.05 indicates differences between normal and abnormal groups.

We examined changes in HRV across pregnancy, parturition, and postpartum periods using time-domain parameters. The SDRR showed significant main effects of period and group (Period: F[2, 16] = 19.5, *p* < 0.05, Group: F[1, 8] = 12.7, *p* < 0.001, repeated measures two-way ANOVA), along with a significant interaction between period and group (F[2, 16] = 8.6, *p* < 0.05). In the normal group, Bonferroni *post hoc* analysis revealed that SDRR during the postpartum period was significantly higher than that during pregnancy (*p* < 0.001) and parturition periods (*p* < 0.01; Fig. 2A). Additionally, SDRR in the abnormal group was significantly lower than that in the normal group during the postpartum period (*p* < 0.01; Fig. 2A). Similarly, RMSSD exhibited significant main effects of period and group (Period: F[2, 16] = 29.0, *p* < 0.0001, Group: F[1, 8] = 8.8, *p* < 0.05), and a significant interaction between period and group (F[2, 16] = 15.3, *p* < 0.01). *Post hoc* analysis revealed that RMSSD in the normal group was significantly higher during the postpartum period than during pregnancy (*p* < 0.001) and parturition periods (*p* < 0.001, Fig. 2B). RMSSD in the abnormal group was significantly lower than that in the normal group during the postpartum period (*p* < 0.01, Fig. 2B).

For CVRR, two-way repeated measures ANOVA showed significant main effects of period and group (Period: F[2, 16] = 20.9, *p* < 0.01, Group: F[1, 8] = 13.7, *p* < 0.01), and a significant interaction between period and group (F[2, 16] = 9.2, *p* < 0.01). *Post hoc* analysis indicated that CVRR in the normal group was significantly higher during the postpartum period than during pregnancy (*p* < 0.001) and parturition periods (*p* < 0.001, Fig. 2C). CVRR in the abnormal group was significantly lower than that in the normal group during the postpartum period (*p* < 0.01, Fig. 2C). These results demonstrate that SDRR, RMSSD, and CVRR significantly increased from pregnancy to postpartum periods in the normal group. However, in the abnormal group, these parameters remained similar to their pre-parturition levels during the postpartum period, in contrast to the dynamic changes observed in the normal group.

### Potential for predicting “abnormal” mice using HRV parameters

Given the differences in autonomic nervous activity between normal and abnormal groups, we hypothesized that these characteristics could be used to predict group membership. To test this hypothesis, we conducted Bayesian logistic regression modeling, which is suitable for small sample sizes. During the pregnancy phase, RMSSD, SDRR, and CVRR did not successfully distinguish between normal and abnormal groups (Bayes Factor (BF) = 3.4, R2 = 0.0; Table S1). However, during the parturition phase, all three parameters successfully modeled the distinction between normal and abnormal groups (BF = 7.6, R2 = 1.0; Table S1). When data from both pregnancy and parturition phases were combined, RMSSD, SDRR, and CVRR failed to model the distinction (BF = 2.5, R2 = 1.0; Table S1). These results suggest that autonomic nervous activity during the parturition phase may help to identify mothers with abnormal parturition or maternal behaviors.

## Discussion

In the normal group, no significant changes in BPM or RR interval were observed from pregnancy to parturition. However, RMSSD, SDRR, and CVRR significantly increased during the postpartum period, indicating that autonomic nervous activity dynamically increased after parturition in normal mice. Unlike previous cross-sectional studies (Klinkenberg et al., 2009; Musa et al., 2017) and studies comparing autonomic activity only before and after parturition (Nagel et al., 2014; Papadopoulos et al., 2021; Uçar, Bülbül & Yıldız, 2022; Felici et al., 2023), our longitudinal study tracking individual mice revealed dynamic autonomic activity across the critical periods surrounding parturition. These changes are likely related to systemic vasodilation occurring postpartum as the body transitions back to a non-pregnant state (Kodogo, Azibani & Sliwa, 2019; Brown et al., 2021; Chauhan & Tadi, 2022; Sarhaddi et al., 2022). Increases in autonomic nervous activity may also be explained by maternal behaviors, such as nursing or physical contact with pups, which affect autonomic function (Ohmura et al., 2023). Notably, similar dynamic changes in RMSSD have been reported in cows, where RMSSD values 24 h after parturition were higher than those in earlier periods (Kovács et al., 2015). This consistency across species suggests that such autonomic nervous activity dynamics represent a shared physiological response.

Notably, we observed a high rate of abnormalities, with 5 out of 10 mice exhibiting abnormal parturition or maternal behaviors, whereas the duration of parturition and the number of pups born did not differ significantly between normal and abnormal groups. These findings suggest that abnormalities may reflect pregnancy-related or underlying factors rather than physical damage during parturition. Stress during pregnancy is a known contributor to abnormal parturition (Coussons-Read, 2013; Lilliecreutz et al., 2016; Garcia-Flores et al., 2020) and maternal behaviors (Zoubovsky et al., 2020; Luft et al., 2022). Acute stress induced by the telemetry device implantation surgery may have contributed to differences between groups. Variations in stress resilience among individuals (Ebner & Singewald, 2017) could explain why some mice experienced normal parturition and caregiving whereas others exhibited abnormalities. However, we did not analyze whether acute stress caused the observed abnormalities; thus, further research is required to determine the effects of stress on parturition and maternal behavior.

In the abnormal group, as in the normal group, no significant changes were observed in BPM or RR intervals before and after parturition. However, unlike the normal group, RMSSD, SDRR, and CVRR in the abnormal group did not increase during the postpartum period. This suggests that the abnormal group experienced lower autonomic nervous activity within 24 h postpartum than the normal group. As the vagus nerve plays a key role in parturition (Brauer & Smith, 2015; Reyes-Lagos et al., 2019), reduced autonomic dynamics may be associated with parturition failure. Furthermore, dams that committed cannibalism/infanticide (on PD1 and PD13), dams that caused all their pups to die through neglect (by PD2), and dams exhibiting parturition failure all exhibited low postpartum autonomic nervous activity. Reduced cardiac autonomic activity, as reflected by SDRR, RMSSD, and CVRR, is associated with stress and mental health conditions such as depression and anxiety (Park et al., 2018; Shea et al., 2008; Kurita et al., 2013; Kimmel et al., 2021; Parisi et al., 2024). Therefore, reduced cardiac autonomic activity in the abnormal group may reflect underlying physiological or emotional factors.

Autonomic nervous activity may help to identify normal and abnormal maternal outcomes. Previous research indicated that autonomic nervous activity can predict maternal emotional states (Li et al., 2022), mental stress (Sharifi-Heris et al., 2023), and postpartum depression (Singh Solorzano, Violani & Grano, 2022). Consistent with these studies, our findings indicate that RMSSD, SDRR, and CVRR values during parturition can distinguish between normal and abnormal dams. This suggests that monitoring autonomic nervous activity around parturition may help to characterize physiological differences between dams with successful and unsuccessful maternal outcomes. However, because of the small sample size in this pilot study, further research is necessary to validate the applicability of our predictive model.

### Limitations of the study

This study provides longitudinal ECG data before, during, and 24 h after parturition and shows that autonomic nervous activity differs between normal and abnormal maternal outcomes. However, several limitations should be considered. First, this study was exploratory and based on a small sample size with *post hoc* group classification into normal and abnormal groups. Because each abnormal outcome (parturition failure, neglect, and cannibalism/infanticide) occurred in only one or two dams, we did not attempt to compare these subtypes and instead treated them as a single group. Therefore, our results should not be interpreted as indicating subtype-specific autonomic patterns, and larger studies are required to address this issue. Second, several potential stressors could have influenced physiological and behavioral outcomes, including transport of pregnant animals, surgical implantation of the telemetry device during pregnancy, and the absence of analgesia after surgery. In addition, detailed background information such as exact age and parity was not available. Although we attempted to minimize variability by obtaining all animals from the same supplier at the same time and performing surgery and housing under standardized conditions, the possible effects of these factors cannot be completely excluded. Third, autonomic nervous activity was assessed only indirectly using ECG-derived HRV parameters. Because we did not perform interventions such as autonomic blockade or parallel physiological measurements, we could not establish a causal relationship between autonomic activity and maternal outcomes. Future studies combining longitudinal monitoring with experimental manipulation are required to clarify the underlying mechanisms. Despite these limitations, this study makes an important contribution to the field by longitudinally tracking individual dams across pregnancy, parturition, and the postpartum period using a noninvasive telemetry approach. Under relatively controlled conditions, we successfully used ECG-derived indices to distinguish between dams with successful and unsuccessful maternal outcomes. Thus, this work provides a methodological and conceptual foundation for future larger-scale and mechanistic studies.

## Conclusions

Our study in awake C57BL/6J mice demonstrates that normal dams experienced dynamic increases in autonomic nervous activity, particularly within 24 h postpartum. Conversely, dams lacking such dynamic changes were more likely to experience parturition failure and develop abnormal maternal behaviors; these outcomes were associated with autonomic nervous activity during parturition. Although HRV analysis only indirectly reflects autonomic function, this study provides a foundation for future research exploring the causal relationships between autonomic nervous activity and maternal state; for example, by directly manipulating autonomic nervous activity. Future research that builds on this foundation can improve our understanding of dynamic changes in autonomic nervous activity and help develop interventions to prevent and mitigate abnormal maternal behaviors (*e.g.*, neglect and cannibalism/infanticide) in various species. Thus, our findings provide an important bridge linking individual differences among mothers to their underlying autonomic nervous function.

## Supplemental Information

10.7717/peerj.21142/supp-1 Supplemental Information 1 Individual mouse reproductive and HRV data

10.7717/peerj.21142/supp-2 Supplemental Information 2 Time schedule in the experiment The experimental timeline. Home cage behavior recording began on GD14, while ECG recording started on GD17.

10.7717/peerj.21142/supp-3 Supplemental Information 3 ECG data from pregnancy to postpartum period (A) Representative continuous ECG trace ( - 30 s) from one mouse. (B)Noise observed in ECG data during parturition. (C) Example of a 5 s continuous ECG recording during the pregnancy period. (D) Example of a 5 s continuous ECG recording during parturition. (E) Example of a 5 s continuous ECG recording during the postpartum period.

10.7717/peerj.21142/supp-4 Supplemental Information 4 BPM and RR interval from pregnancy to postpartum period (A) The average heart rate (BPM) across the pregnancy to postpartum period. No significant differences were observed between the normal group (black) and the abnormal group (red: □ = parturition failure, ×= neglect, + = cannibalism/infanticide ) by two-way repeated measures ANOVA with Bonferroni test: Group (F[1,8] = 3.3, *p* = 0.11), Period (F[2,16] = 2.8, *p* = 0.091), Group × Period (F [2,16] = 8.6, *p* = 0.14). (B) The average RR interval across the pregnancy to postpartum period. No significant differences were observed between the normal group (black) and the abnormal group (red: □ = parturition failure, × = neglect, + = cannibalism/infanticide ) by two-way repeated measures ANOVA with Bonferroni test: Group (F[1,8] = 2.0, p = 0.17), Period (F[2,16] = 3.8, *p* = 0.09), Group × Period (F [2,16] = 3.1, *p* = 0.07).

10.7717/peerj.21142/supp-5 Supplemental Information 5 Bayesian logistic regression of autonomic nervous activity during pregnancy and parturition phases The posterior estimates for the Bayesian logistic regression model assessing autonomic nervous activity during pregnancy and parturition. The variables RMSSD, SDRR, and CVRR were used as predictors to distinguish between normal and abnormal groups. The table includes inclusion Bayes factor (BFinclusion) and 95% credible intervals for each variable.

10.7717/peerj.21142/supp-6 Supplemental Information 6 ARRIVE checklist

10.7717/peerj.21142/supp-7 Supplemental Information 7 The Onset of Parturition This video shows a female mouse beginning to give birth. The video is presented at twice the real-time speed.

10.7717/peerj.21142/supp-8 Supplemental Information 8 Representative ECG recording from an awake mouse Representative continuous ECG trace (-30 s) recorded from an awake mouse, demonstrating stable signal quality.

## Additional Information and Declarations

### Data Availability

The following information was supplied regarding data availability: The raw data is available in the Supplemental File.
