# Supplementary figures and images for: Dynamic changes in postpartum autonomic nervous system in normal mice: a pilot study
Source: PeerJ. 2026 May 18;14:e21142. doi: 10.7717/peerj.21142 (PMC13192457; doi:10.7717/peerj.21142)

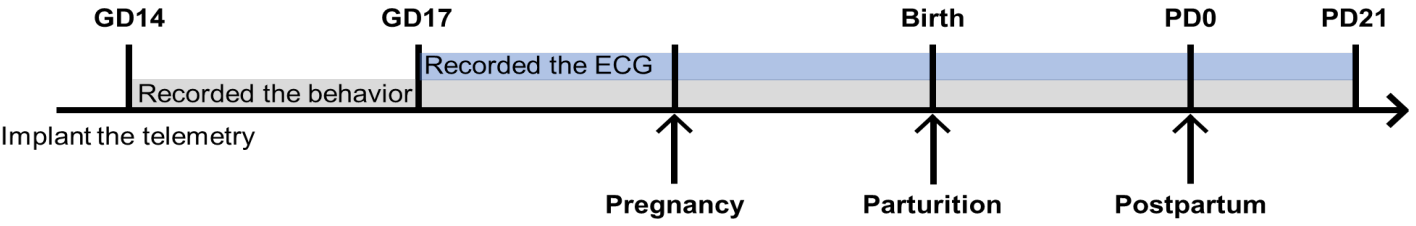

Supplement: Supplemental Information 2 — The experimental timeline. Home cage behavior recording began on GD14, while ECG recording started on GD17. [file peerj-14-21142-s002.pdf]

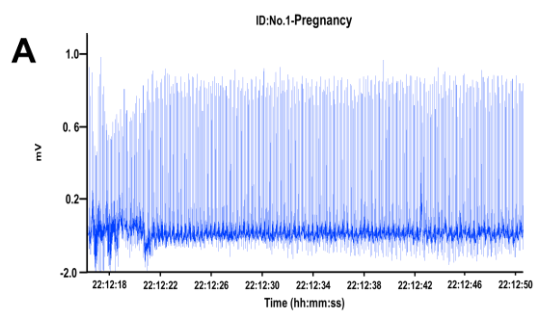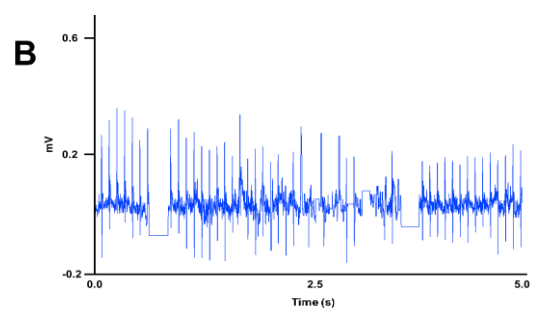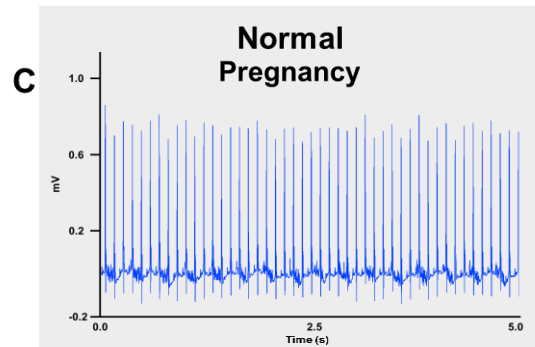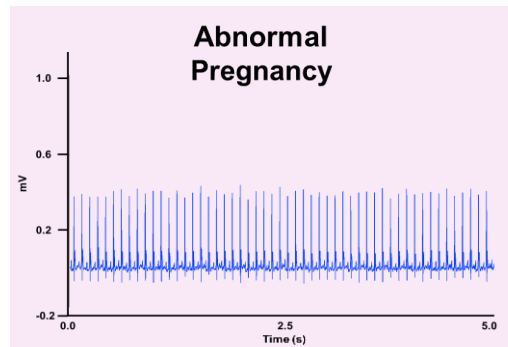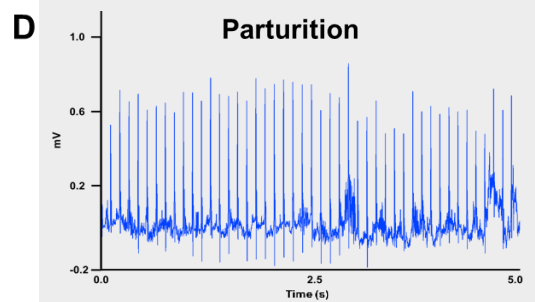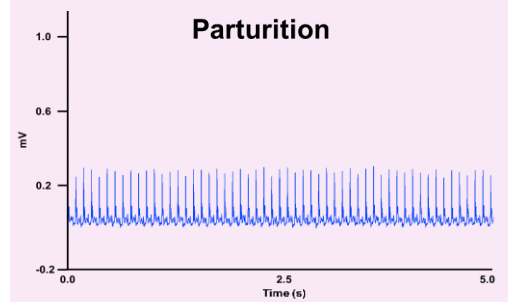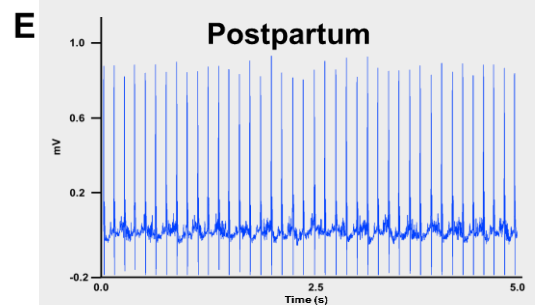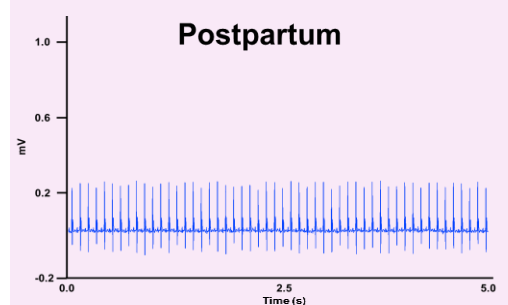

Supplement: Supplemental Information 3 — (A) Representative continuous ECG trace ( - 30 s) from one mouse. (B)Noise observed in ECG data during parturition. (C) Example of a 5 s continuous ECG recording during the pregnancy period. (D) Example of a 5 s continuous ECG recording during parturition. (E) Example of a 5 s continuous ECG recording during the postpartum period. [file peerj-14-21142-s003.pdf]

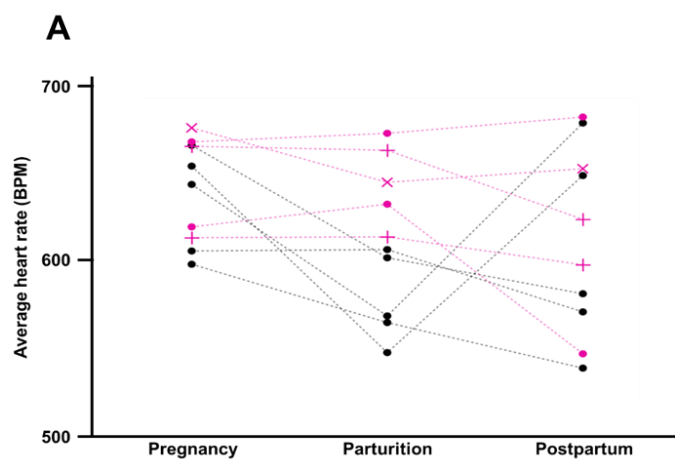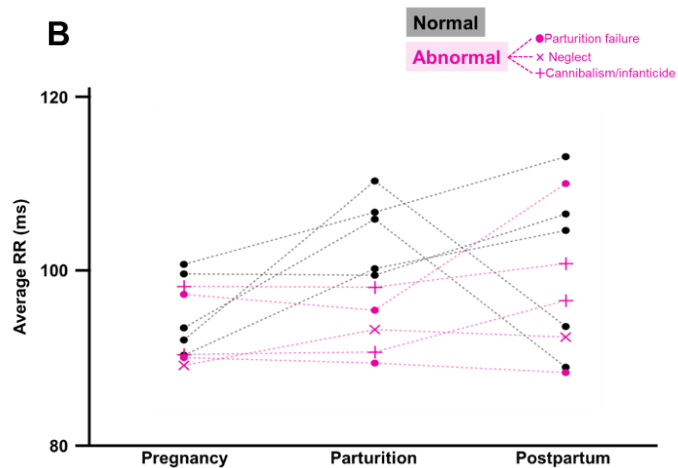

Supplement: Supplemental Information 4 — (A) The average heart rate (BPM) across the pregnancy to postpartum period. No significant differences were observed between the normal group (black) and the abnormal group (red: □ = parturition failure, ×= neglect, + = cannibalism/infanticide ) by two-way repeated measures ANOVA with Bonferroni test: Group (F[1,8] = 3.3, p = 0.11), Period (F[2,16] = 2.8, p = 0.091), Group × Period (F [2,16] = 8.6, p = 0.14). (B) The average RR interval across the pregnancy to postpartum period. No significant differences were observed between the normal group (black) and the abnormal group (red: □ = parturition failure, × = neglect, + = cannibalism/infanticide ) by two-way repeated measures ANOVA with Bonferroni test: Group (F[1,8] = 2.0, p = 0.17), Period (F[2,16] = 3.8, p = 0.09), Group × Period (F [2,16] = 3.1, p = 0.07). [file peerj-14-21142-s004.pdf]

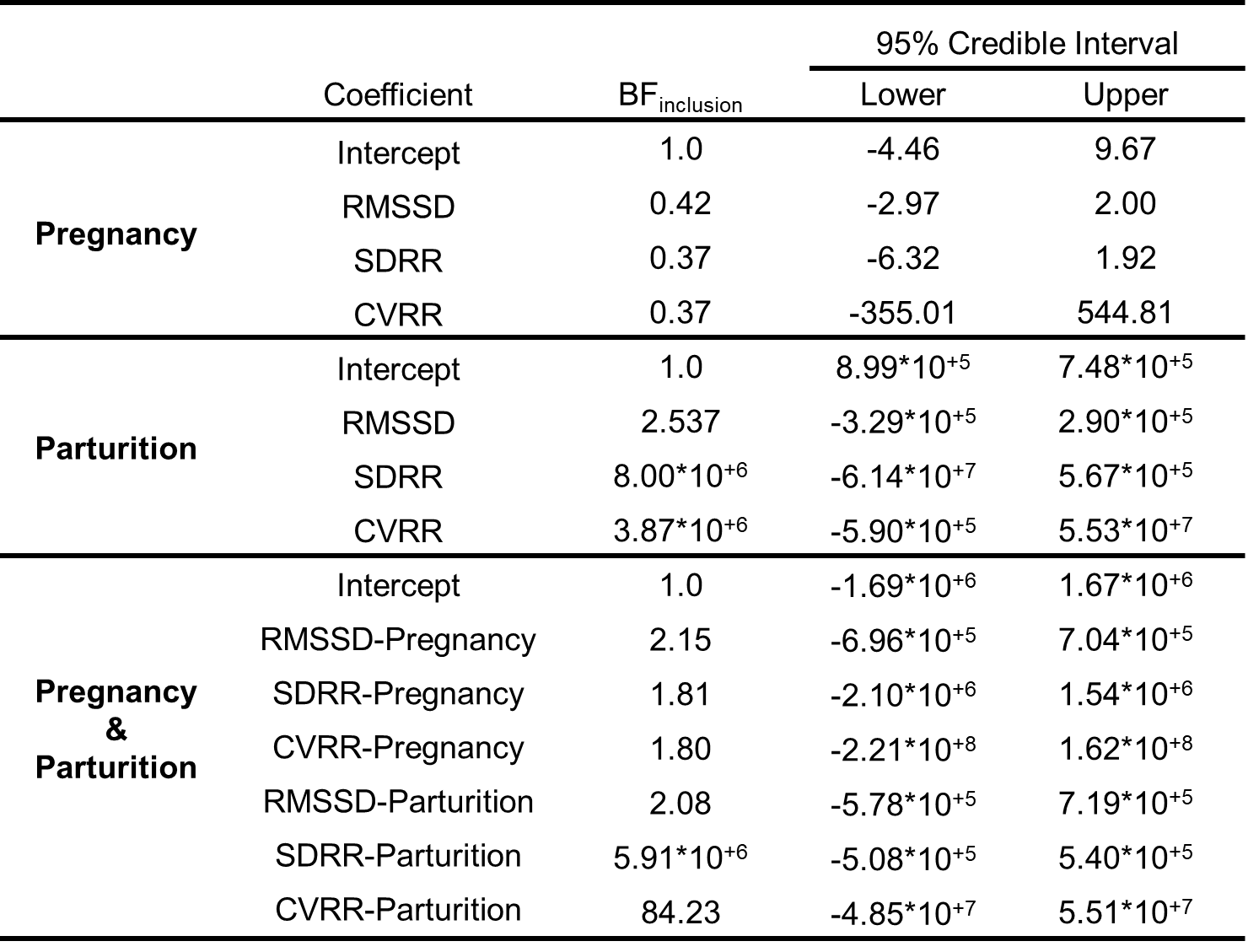

Supplement: Supplemental Information 5 — The posterior estimates for the Bayesian logistic regression model assessing autonomic nervous activity during pregnancy and parturition. The variables RMSSD, SDRR, and CVRR were used as predictors to distinguish between normal and abnormal groups. The table includes inclusion Bayes factor (BFinclusion) and 95% credible intervals for each variable. [file peerj-14-21142-s005.docx]
